# Associations of plasma angiostatin and amyloid-β and tau levels in Alzheimer’s disease

**DOI:** 10.1038/s41398-022-01962-6

**Published:** 2022-05-10

**Authors:** Yuan Cheng, Jun-Rong Ren, Jie-Ming Jian, Chen-Yang He, Man-Yu Xu, Gui-Hua Zeng, Cheng-Rong Tan, Ying-Ying Shen, Wang-Sheng Jin, Dong-Wan Chen, Hui-Yun Li, Xu Yi, Yuan Zhang, Xian-Le Bu, Yan-Jiang Wang

**Affiliations:** 1 Department of Neurology and Centre for Clinical Neuroscience, Daping Hospital, Third Military Medical University, Chongqing, China; 2 Institute of Brain and Intelligence, Third Military Medical University, Chongqing, China; 3 Chongqing Key Laboratory of Ageing and Brain Diseases, Chongqing, China; 4 Center for Excellence in Brain Science and Intelligence Technology, Chinese Academy of Sciences, Shanghai, China

**Keywords:** Diagnostic markers, Physiology, Biomarkers

## Abstract

Angiostatin, an endogenous angiogenesis inhibitor generated by the proteolytic cleavage of plasminogen, was recently reported to contribute to the development of Alzheimer’s disease (AD). However, whether there are pathological changes in angiostatin levels in individuals with AD dementia is unclear, and whether plasma angiostatin has a relationship with major AD pathological processes and cognitive impairment remains unknown. To examine plasma angiostatin levels in patients with AD dementia and investigate the associations of angiostatin with blood and cerebrospinal fluid (CSF) AD biomarkers, we conducted a cross-sectional study including 35 cognitively normal control (CN) subjects and 59 PiB-PET-positive AD dementia patients. We found that plasma angiostatin levels were decreased in AD dementia patients compared to CN subjects. Plasma angiostatin levels were negatively correlated with plasma Aβ42 and Aβ40 levels in AD dementia patients and positively correlated with CSF total tau (t-tau) levels and t-tau/Aβ42 in AD dementia patients with APOE-ε4. In addition, plasma angiostatin levels had the potential to distinguish AD from CN. These findings suggest a link between angiostatin and AD pathogenesis and imply that angiostatin might be a potential diagnostic biomarker for AD.

## Introduction

Alzheimer’s disease (AD) is the most common neurodegenerative disease in the world [1]. The main pathological hallmarks of AD include extracellular senile plaques consisting of β-amyloid (Aβ) and intracellular neurofibrillary tangles composed of hyperphosphorylated tau [2]. Several risk factors, including aging, neuroinflammation, and vascular dysfunction, have been considered to contribute to AD pathogenesis [3–6]. However, the pathogenic mechanisms underlying AD have not yet been elucidated.

In recent years, accumulating evidence has indicated that abnormal angiogenesis may participate in the pathogenesis of AD [7, 8]. Disruption of pro-and antiangiogenic processes has been considered to directly contribute to AD pathology [9]. Development of the blood-brain barrier (BBB) is initiated during angiogenesis, and BBB integrity plays a vital role in brain homeostasis and neuroprotection [10]. Disruption of the BBB has been shown to coincide with the onset of cognitive impairment [11, 12]. In addition, modulating cerebrovascular neoangiogenesis could reverse brain pathology in a preclinical model of AD, which strengthens the link between angiogenesis and AD [13].

Angiostatin, an endogenous angiogenesis inhibitor that is cleaved from plasminogen, may be involved in the pathogenesis of AD. In a rat model of AD, treatment with angiostatin significantly reduced microgliosis, diminished microvessels, and improved neuronal viability [14]. In addition, physical exercise ameliorated cognitive impairment in an AD rat model by modulating angiostatin levels [15]. Recent large-scale plasma proteomic analysis showed that angiostatin was associated with dementia risk [16]. However, there are few clinical studies on the relationship between angiostatin and AD biomarkers. Our study aimed to explore alterations in plasma angiostatin levels in cognitively normal control (CN) subjects and AD dementia patients and the correlations of plasma angiostatin levels with brain and blood AD biomarkers.

## Materials and methods

### Study population

All participants were recruited from the Chongqing Ageing & Dementia Study (CADS) [17], which is an ongoing cohort study initiated in 2010 that aimed to explore the mechanisms of the evolution of aging in AD to identify biomarkers for early diagnosis and the development of interventional strategies for AD.

AD dementia patients were recruited from the Neurology Department of Daping Hospital from December 2018 to May 2020. The clinical assessment of AD dementia was performed following a previously used protocol [18]. In brief, the demographic data and medical history (such as hypertension, coronary heart disease, and diabetes mellitus) were collected. The cognitive and functional status of participants with memory and cognitive complaints was assessed using a neuropsychological battery that included the Mini-Mental State Examination (MMSE), Montreal Cognitive Assessment, activities of daily living, auditory verbal learning test, clock drawing test, Trail Making Test, Boston naming test, digit span test, clinical dementia rating, Pfeiffer Outpatient Disability Questionnaire, and Hachinski ischemic score. The clinical diagnosis of AD was based on the criteria of the National Institute of Neurological and Communicative Diseases and Stroke/AD and Related Disorders Association following the protocols we used before [19] and contained the following steps: (1) insidious onset of symptoms, (2) clear history of cognitive deterioration, and (3) significant cognitive impairments in at least one of the following categories: visuospatial presentation, language presentation, amnestic presentation, or executive dysfunction. (4) Individuals with familial AD and other types of dementia were excluded. The clinically diagnosed AD patients were further evaluated with Aβ positron emission tomography (PET) scanning with PiB to detect and quantify Aβ deposition in the brain. All the subjects enrolled in the AD dementia group were amyloid positive and referred to as AD dementia patients [19].

Age- and sex-matched cognitively normal control (CN) subjects had no history or signs of neurological disorders in the clinical examination and were randomly recruited from the hospital at the same time. CN subjects with CSF Aβ42 ≤ 930.35 pg/mL who met the preclinical diagnostic criteria of the CADS were excluded.

Subjects were excluded for the following reasons: (1) a family history of dementia; (2) a concomitant neurological disorder that could potentially affect the cognitive function or other types of dementia; (3) severe cardiac, pulmonary, hepatic, or renal diseases or any type of tumor; (4) enduring mental illness (e.g., schizophrenia); (5) diseases that may affect coagulation and the fibrinolytic system (e.g., bleeding disorders); (6) recently used treatments that affect angiostatin levels (e.g., blood transfusion); and (7) refusal to participate in the study.

Finally, a total of 35 CN participants and 59 AD dementia patients were included in the present study. Written consent was obtained from all participants or their legal representatives. This study was approved by the Institutional Review Board of Daping Hospital.

### Sample collection

Fasting blood was collected from all participants between 07:00 and 09:00. The blood samples were centrifuged within an hour of collection, and ethylenediaminetetraacetic acid (EDTA) plasma was aliquoted into 0.5 mL polypropylene tubes and stored at −80 °C. Cerebrospinal fluid (CSF) samples were collected by lumbar puncture from 35 CN subjects and 25 AD dementia patients. The CSF samples were centrifuged at 2000 x *g* at 4 °C for 10 min, and the aliquots were then immediately frozen and stored at −80 °C until use. Informed consent was obtained before the acquisition of the blood and CSF samples.

### PET acquisition and analysis

Subjects were asked to fast for at least 6 h but had free access to water before the PET scan. PET scans were performed with a Siemens Biograph 64 PET/CT machine (Siemens, Munich, Germany) in three-dimensional mode. PiB-PET scans were performed according to standardized research protocols [20] in separate sessions. Specifically, a dynamic 90-min emission scan was performed following intravenous injection of ^11^C-PiB after 10 min of a transmission scan. Standardized images were extracted within the regulated interval time after injection. All scans were performed in a dimly lit and quiet room with subjects in a resting state. The visual ratings were performed by radiologists with specific training in the interpretation of PET who were blinded to all clinical information, and the final ratings were decided by consensus. PiB-PET scans were rated as either PiB-PET^+^ (binding in at least one cortical brain region, such as frontal, temporal, parietal, or occipital) or PiB-PET^−^ (binding predominantly in white matter) [21].

### Measurements of plasma angiostatin, Aβ, and tau levels

Angiostatin levels in plasma were determined using human angiostatin enzyme-linked immunosorbent assay (ELISA) kits (Jiangsu Jingmei Biotechnology Co., Ltd., Yancheng, China). Plasma levels of Aβ42 and Aβ40 were measured using the commercially available single-molecule array (SIMOA) Human Neurology 3-Plex A assay kit (Quanterix, United States) using the automated SIMOA HD-1 zanalyzer (Quanterix, United States). CSF levels of Aβ40, Aβ42, total tau (t-tau), and phosphorylated tau-181 (p-tau) were measured using human Aβ and tau ELISA kits (Innotest, United States) [18].

### Statistical analysis

Demographic characteristics with continuous variables are described as the median/mean, and categorical data are summarized as absolute frequencies. Differences in the frequencies of sex and apolipoprotein E (APOE)-ε4 categories were assessed by chi-square tests. Differences in other demographic characteristics and angiostatin levels were tested using a two-sample independent *t* test (or Student’s *t* test with Welch’s correction if the *F* test showed significantly different variances between groups). The correlations of angiostatin levels with Aβ and tau levels were analyzed by partial correlation analyses adjusted by age, sex, education level, and APOE-ε4 genotype. The sample size in the current study has been estimated using a professional tool (http://www.powerandsamplesize.com/) to ensure a desired power. The data are expressed as the mean ± standard deviation (SD). All hypothesis testing was two-sided, and *p* < 0.05 was defined as statistically significant. The computations were performed with SPSS version 20.0 (SPSS Inc., United States).

## Results

### Characteristics of the study population

The characteristics of the subjects are shown in Table 1. The study consisted of 59 AD dementia patients with positive PiB-PET and 35 age- and sex-matched CN subjects. There were no significant differences in age, sex, education level, or comorbidities (diabetes mellitus, hypertension, and cardiovascular disease) between the AD and CN groups. The AD dementia group had a higher percentage of APOE-ε4 carriers (*p* < 0.0001). The mean MMSE score of the AD dementia group was significantly lower than that of the normal control group (*p* < 0.0001).

**Table 1:** Characteristics of all participants.

Characteristics	CN (*N* = 35)	AD dementia (*N* = 59)	*p* values
Age, mean (SD), y	67.46 (5.79)	66.88 (10.86)	0.7727
Female, *N* (%)	16 (45.71)	33 (55.93)	0.3377
Education level, mean (SD), y	9.31 (4.14)	9.42 (4.30)	0.9040
MMSE score, mean (SD)	26.88 (2.07)	14.51 (6.84)	<0.0001
APOE-ε4 carriers, N (%)	8 (22.86)	32 (54.24)	<0.0001
Diabetes (%)	4 (11.43)	9 (15.90)	0.7610
Hypertension (%)	6 (17.12)	13 (22.30)	0.6082
Coronary artery disease (%)	8 (22.86)	12 (20.30)	0.7986
Stroke history (%)	3 (8.57)	4 (7.30)	>0.9999

The results are shown as the mean (SD) or number (%).

*CN* cognitively normal control, *AD* Alzheimer’s disease, *y* years, *MMSE* Mini-Mental State Examination, *APOE* apolipoprotein E, *N* number, *SD* standard deviation.

### Comparisons of plasma angiostatin levels between the control and AD groups

**Fig. 1:**
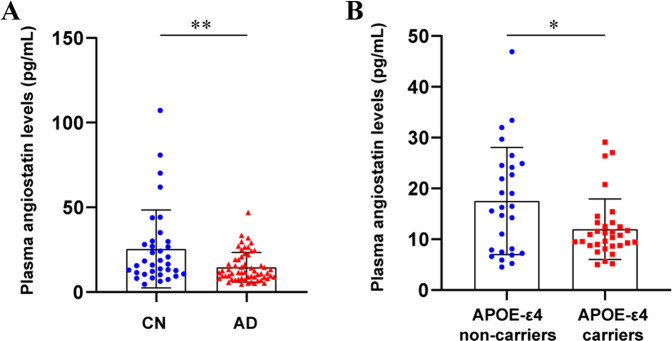
Comparison of plasma angiostatin levels between different groups. Comparison of plasma angiostatin levels between the cognitively normal controls (CN) and PiB-PET^+^ AD dementia patients (**A**). Comparison of the plasma angiostatin levels between APOE-ε4 carriers and noncarriers among the PiB-PET^+^ AD dementia patients (**B**).

Plasma angiostatin levels in patients with PiB-PET^+^ AD dementia were lower than those in the CN subjects (14.52 ± 8.75 vs. 25.41 ± 22.98 pg/ml, *p* = 0.0015) (Fig. 1A). Plasma angiostatin concentrations were lower in APOE-ε4 carriers than in APOE-ε4 noncarriers among the patients with AD dementia (12.00 ± 5.96 pg/ml vs. 17.51 ± 10.55, *p* = 0.0145) (Fig. 1B).

### Correlations of plasma angiostatin with plasma Aβ levels

**Fig. 2:**
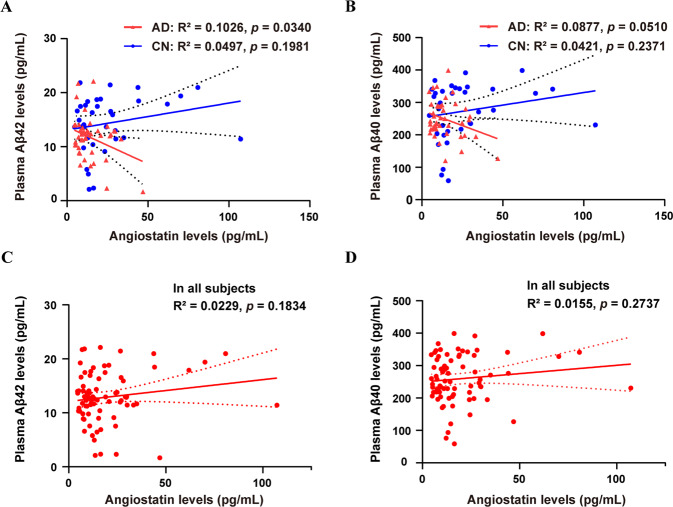
Correlation analyses of plasma angiostatin with plasma Aβ levels. Correlations of angiostatin levels with plasma Aβ42 levels (**A**) and Aβ40 levels (**B**) in PiB-PET^+^ AD dementia patients and cognitively normal controls (CN). Correlations of angiostatin levels with plasma Aβ42 levels (**C**) and Aβ40 levels (**D**) in all participants.

There were 35 CN subjects and 44 AD dementia patients among the participants who had plasma Aβ levels measured. Plasma angiostatin levels were negatively correlated with plasma Aβ42 levels (R^2^ = 0.1026, *p* = 0.034; Fig. 2A), and there was a tendency towards a negative correlation with plasma Aβ40 levels (R^2^ = 0.0877, *p* = 0.051; Fig. 2B) in PiB-PET^+^ AD dementia patients. There were no correlations between angiostatin levels and plasma Aβ42 or Aβ40 levels in either CN subjects or all participants (Fig. 2C, D). In addition, there was no correlation of angiostatin levels with age or MMSE scores across all participants (Supplementary Fig. 1).

### Correlations of plasma angiostatin levels with CSF Aβ and tau levels

**Fig. 3:**
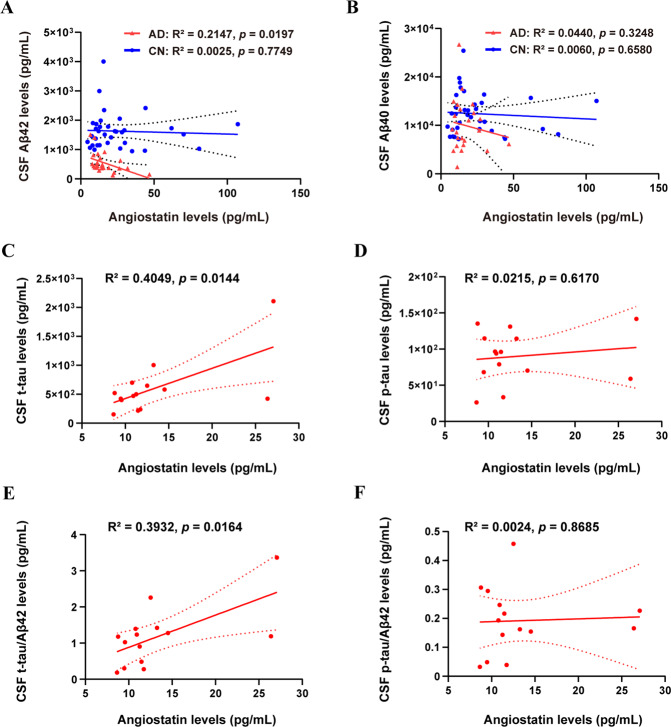
Correlations between plasma angiostatin levels and CSF biomarkers. Correlations of angiostatin levels with CSF Aβ42 levels (**A**) and Aβ40 levels (**B**) in PiB-PET^+^ AD dementia patients and cognitively normal controls (CN). Correlations of angiostatin levels with t-tau levels (**C**) and p-tau levels (**D**), t-tau/Aβ42 (**E**), and p-tau/Aβ42 (**F**) in PiB-PET^+^ AD dementia patients with APOE-ε4.

To further reveal the relationships between angiostatin and AD pathological changes, we analyzed the correlations of plasma angiostatin with CSF Aβ, t-tau, and p-tau levels. Among all enrolled subjects, CSF Aβ was measured in 35 CN subjects and 25 AD dementia patients. Plasma angiostatin levels showed a negative correlation with CSF Aβ42 levels in AD dementia patients (R^2^ = 0.2147, *p* = 0.0197) but had no correlation with CSF Aβ40 (*p* = 0.3248) (Fig. 3A, B), Aβ42/40 (*p* = 0.3913), Aβ42/t-tau (*p* = 0.3540), or Aβ42/p-tau (*p* = 0.4677). No significant correlation between angiostatin levels and CSF Aβ levels was found in the CN group. In addition, t-tau and p-tau levels had no correlations with plasma angiostatin levels in either AD or CN groups (data not shown). Then, we performed subgroup analyses between APOE-ε4 carriers and noncarriers among those in the AD group and found that plasma angiostatin levels had a positive correlation with CSF t-tau levels (R^2^ = 0.4049, *p* = 0.0144) but did not correlate with CSF p-tau (*p* = 0.6170) (Fig. 3C, D). Based on the notion that the ratio of CSF tau to Aβ42 has a higher correlation with brain Aβ deposition [22, 23], we analyzed the correlations of plasma angiostatin with these ratios. Plasma angiostatin levels had a positive correlation with CSF t-tau/Aβ42 (R^2^ = 0.3932, *p* = 0.0164) in AD APOE-ε4 carriers but showed no correlation with CSF p-tau/Aβ42 (*p* = 0.8685) (Fig. 3E, F).

### Diagnostic value of plasma angiostatin levels for AD

**Fig. 4:**
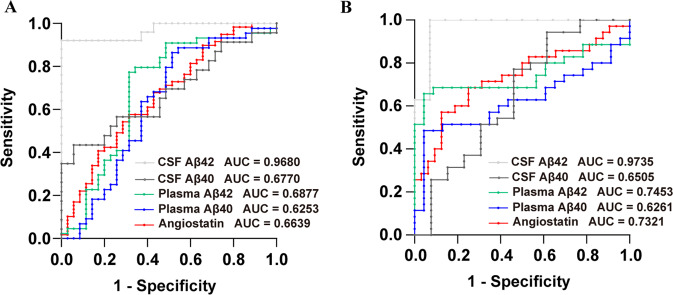
Receiver operating characteristic (ROC) curves of angiostatin. ROC curves of angiostatin and common biomarkers of AD. Diagnostic value of angiostatin and known biomarkers in distinguishing AD patients from cognitively normal controls (**A**). Diagnostic value of angiostatin and known biomarkers in distinguishing AD patients with APOE-ε4 from cognitively normal controls (**B**).

To discriminate between AD and CN subjects, the area under the curve (AUC) for plasma angiostatin was 0.6639 (*p* = 0.0081, 95% CI = 0.5491–0.7787), which was slightly higher than 0.6253 (*p* = 0.0568, 95% CI = 0.4938–0.7569) for plasma Aβ40 and lower than 0.6877 (*p* = 0.0043, 95% CI = 0.5610–0.8143) for plasma Aβ42 and 0.6770 (*p* = 0.0235, 95% CI = 0.5273–0.8267) for CSF Aβ40. The AUC of CSF Aβ42 was high at 0.9680 (*p* < 0.0001, 95% CI = 0.9227–1.000) (Fig. 4A). Then, we analysed the diagnostic value of angiostatin in distinguishing AD patients with APOE-ε4 from CN subjects, and the AUC for plasma angiostatin increased to 0.7321 (*p* = 0.0011, 95% CI = 0.6102–0.8541), which was still higher than that for the plasma biomarker Aβ40 (AUC = 0.6261) but still lower than that for Aβ42 (AUC = 0.7453) (Fig. 4B). These pieces of evidence suggested that the diagnostic efficiency of plasma angiostatin was higher in AD dementia patients with positive APOE-ε4.

## Discussion

The current study first demonstrated that plasma angiostatin levels are lower in AD dementia patients than in CN subjects and explored the correlations between angiostatin levels and Aβ and tau levels in humans. In AD dementia patients, plasma angiostatin levels were negatively correlated with plasma and CSF Aβ42 levels and positively correlated with CSF t-tau and t-tau/Aβ42 levels in APOE-ε4 + AD dementia patients but did not correlate with CSF p-tau levels, even in the APOE-ε4 carriers. The results also showed that plasma angiostatin has a potential diagnostic value for distinguishing AD patients from CN subjects.

A recent study suggested that higher levels of plasma angiostatin were associated with lower dementia risk in the Atherosclerosis Risk in Communities (ARIC) cohort [16]. However, the following Mendelian randomization results imply that a genetic propensity for greater plasma angiostatin levels increases AD risk [16]. The relationship between blood angiostatin levels and AD is controversial. In addition, a previous study found that Aβ could promote extensive neoangiogenesis leading to increased hypervascularization [24], while the cerebral microvasculature was shown to be reduced around Aβ plaques [25]. Hence, the role of Aβ in angiogenesis and how angiogenesis may contribute to the pathogenesis of AD remain unclear.

Angiostatin, an endogenous angiogenesis inhibitor, can block the growth of new blood vessels via a negative feedback homeostatic process [26]. Thus, angiostatin can be considered a homeostatic mechanism to limit angiogenesis. However, the enhanced and severe angiogenesis in the AD brain may damage this negative feedback loop. The breakdown of the balance between angiogenesis and vascular inhibition further strengthens the degree of angiogenesis. In this manner, the level of plasma angiostatin may reflect, to some extent, the severity of angiogenesis and AD-related neuropathology in the AD brain. Therefore, the overall levels of plasma angiostatin were lower in AD dementia patients, and they were negatively correlated with plasma and CSF Aβ levels but positively correlated with CSF t-tau levels in AD dementia patients in the current study. A previous study demonstrated that APOE-ε4 carriers showed a much more severe Aβ burden than noncarriers that could lead to excessive angiogenesis [27], which may explain why plasma angiostatin levels were lower in APOE-ε4 carriers than in APOE-ε4 noncarriers among the AD dementia patients. These results suggest that future therapeutics targeting brain angiogenesis also need to consider the APOE-ε4 allele status.

P-tau is considered a specific biomarker for tau pathology, while t-tau is considered a general marker of neurodegeneration. The current study showed that angiostatin was correlated with CSF t-tau but not p-tau, indicating that angiostatin is related to the severity of neurodegeneration in patients with AD dementia. As an indicator reflecting angiogenesis, angiostatin is more likely to directly act on blood vessels, and its effect on neuronal damage may be indirect, which explains some weak correlations in the current study to some extent. There are fewer reports examining angiostatin and tau phosphorylation. More research is warranted to explore the relationship between angiostatin and p-tau in the future.

Indeed, antiangiogenic treatment has been applied in tumor research for some time [28, 29], and angiostatin also seems to be therapeutically promising for AD. A previous study demonstrated that angiostatin can inhibit neuronal loss, reduce inflammation and stabilize vascular remodeling in an animal model of AD [14]. Furthermore, a recent study demonstrated that targeting the proangiogenic pathway using the anticancer drug axitinib, a small molecule tyrosine kinase inhibitor targeting vascular endothelial growth factor receptor (VEGFR), platelet-derived growth factor receptor (PDGFR), and c-KIT receptors, dramatically reduced cerebrovascular angiogenesis and promoted blood-brain barrier integrity, resulting in improved Aβ clearance and diminished brain Aβ burden in a mouse model of AD [13]. This evidence suggests that angiogenic inhibition may have therapeutic potential for AD and that angiostatin may also be useful in AD treatment. Further studies are needed to investigate the therapeutic effect of antiangiogenic interventions for AD.

Although CSF biomarkers already have high sensitivity and specificity in the diagnosis of AD, studies on novel blood biomarkers of AD are still attractive due to their convenience and noninvasiveness in clinical practice. Our study first provides evidence that plasma angiostatin may be a potential biomarker for distinguishing AD dementia patients from CN subjects, and it seems to be more applicable when used in APOE-ε4-positive AD dementia patients. Indeed, APOE-ε4 increases the risk for AD and is also associated with an earlier age of disease onset [30]. Hence, further studies are encouraged to validate the diagnostic value of angiostatin in preclinical AD.

One of the limitations of the present study is that this is a cross-sectional observational study, and whether some of the identified trends in the present study could be generalized to a broader population still needs validation. Mechanistic conclusions cannot be drawn from the present data, and large-scale and multicentre cohort studies are needed to further clarify the associations between AD and angiostatin in the future. Moreover, in some cases, AD diagnosis is made based on clinical symptoms and routine MRI and blood tests according to 1984 criteria. Previous studies found that some AD patients diagnosed by 1984 criteria were negative in Aβ deposition in PET test [31, 32]. Therefore, it is uncertain whether the findings of this study would also be apparent in AD participants diagnosed by criteria without PET or biomarker tests, which needs to be investigated in the future. The current study did not analyze the association between angiostatin and vascular factors that also have an impact on AD. At the same time, whether drugs targeting angiostatin could improve the cognitive function of AD dementia patients remains to be further investigated.

In conclusion, this study provides clinical evidence for the relationship between angiostatin and AD, suggesting that angiostatin may be a potential biomarker for brain angiogenesis in AD, which is worthy of further exploration in the future.

## Supplementary information

Supplemental Information

## References

[1] Wang J, Gu BJ, Masters CL, Wang YJ (2017). A systemic view of Alzheimer disease - insights from amyloid-beta metabolism beyond the brain. Nat Rev Neurol.

[2] Ittner LM, Gotz J (2011). Amyloid-beta and tau-a toxic pas de deux in Alzheimer’s disease. Nat Rev Neurosci.

[3] Karch CM, Goate AM (2015). Alzheimer’s disease risk genes and mechanisms of disease pathogenesis. Biol Psychiatry.

[4] Huang Y, Mucke L (2012). Alzheimer mechanisms and therapeutic strategies. Cell.

[5] Hou Y, Dan X, Babbar M, Wei Y, Hasselbalch SG, Croteau DL (2019). Ageing as a risk factor for neurodegenerative disease. Nat Rev Neurol.

[6] Calsolaro V, Edison P (2016). Neuroinflammation in Alzheimer’s disease: current evidence and future directions. Alzheimer’s Dement: J Alzheimer’s Assoc.

[7] Jefferies WA, Price KA, Biron KE, Fenninger F, Pfeifer CG, Dickstein DL (2013). Adjusting the compass: new insights into the role of angiogenesis in Alzheimer’s disease. Alzheimer’s Res Ther.

[8] Vagnucci AH, Li WW (2003). Alzheimer’s disease and angiogenesis. Lancet.

[9] Lee ST, Chu K, Jung KH, Park HK, Kim DH, Bahn JJ (2009). Reduced circulating angiogenic cells in Alzheimer disease. Neurology.

[10] Profaci CP, Munji RN, Pulido RS, Daneman R (2020). The blood-brain barrier in health and disease: Important unansweredquestions. J Experimental Med.

[11] Zhao M, Jiang XF, Zhang HQ, Sun JH, Pei H, Ma LN (2021). Interactions between glial cells and the blood-brain barrier and their role in Alzheimer’s disease. Ageing Res Rev.

[12] Zhang Q, Xie C, Apolipoprotein E (2021). Drives early blood-brain barrier damage in Alzheimer’s disease. Neurosci Bull.

[13] Singh CSB, Choi KB, Munro L, Wang HY, Pfeifer CG, Jefferies WA (2021). Reversing pathology in a preclinical model of Alzheimer’s disease by hacking cerebrovascular neoangiogenesis with advanced cancer therapeutics. EBioMedicine.

[14] Ryu JK, Little JP, Klegeris A, Jantaratnotai N, McLarnon JG (2013). Actions of the anti-angiogenic compound angiostatin in an animal model of Alzheimer’s disease. Curr Alzheimer Res.

[15] Zarezadehmehrizi A, Hong J, Lee J, Rajabi H, Gharakhanlu R, Naghdi N (2021). Exercise training ameliorates cognitive dysfunction in amyloid beta-injected rat model: possible mechanisms of Angiostatin/VEGF signaling. Metab Brain Dis.

[16] Walker KA, Chen J, Zhang J, Fornage M, Coresh J (2021). Large-scale plasma proteomic analysis identifies proteins and pathways associated with dementia risk. Nat Aging.

[17] Wang J, Fan DY, Li HY, He CY, Shen YY, Zeng GH (2022). Dynamic changes of CSF sPDGFRbeta during ageing and AD progression and associations with CSF ATN biomarkers. Mol Neurodegeneration.

[18] Li WW, Wang Z, Fan DY, Shen YY, Chen DW, Li HY (2020). Association of polygenic risk score with age at onset and cerebrospinal fluid biomarkers of Alzheimer’s disease in a Chinese cohort. Neurosci Bull.

[19] McKhann GM, Knopman DS, Chertkow H, Hyman BT, Jack CR, Kawas CH (2011). The diagnosis of dementia due to Alzheimer’s disease: recommendations from the National Institute on Aging-Alzheimer’s Association workgroups on diagnostic guidelines for Alzheimer’s disease. Alzheimer’s Dement: J Alzheimer’s Assoc.

[20] Lopresti BJ, Klunk WE, Mathis CA, Hoge JA, Ziolko SK, Lu X (2005). Simplified quantification of Pittsburgh Compound B amyloid imaging PET studies: a comparative analysis. J Nucl Med: Off Publ, Soc Nucl Med.

[21] Ossenkoppele R, Prins ND, Pijnenburg YA, Lemstra AW, van der Flier WM, Adriaanse SF (2013). Impact of molecular imaging on the diagnostic process in a memory clinic. Alzheimer’s Dement: J Alzheimer’s Assoc.

[22] Schindler SE, Gray JD, Gordon BA, Xiong C, Batrla-Utermann R, Quan M (2018). Cerebrospinal fluid biomarkers measured by Elecsys assays compared to amyloid imaging. Alzheimer’s Dement: J Alzheimer’s Assoc.

[23] Hansson O, Seibyl J, Stomrud E, Zetterberg H, Trojanowski JQ, Bittner T (2018). CSF biomarkers of Alzheimer’s disease concord with amyloid-beta PET and predict clinical progression: a study of fully automated immunoassays in BioFINDER and ADNI cohorts. Alzheimer’s Dement: J Alzheimer’s Assoc.

[24] Biron KE, Dickstein DL, Gopaul R, Jefferies WA (2011). Amyloid triggers extensive cerebral angiogenesis causing blood brain barrier permeability and hypervascularity in Alzheimer’s disease. PloS ONE.

[25] Alvarez-Vergara MI, Rosales-Nieves AE, March-Diaz R, Rodriguez-Perinan G, Lara-Urena N, Ortega-de San Luis C (2021). Non-productive angiogenesis disassembles Ass plaque-associated blood vessels. Nat Commun.

[26] Kotulska-Kucharz A, Kopec-Medrek M, Kucharz EJ (2018). Serum angiostatin and endostatin levels in patients with granulomatosis with polyangiitis and immune complex small vessel vasculitis. Reumatologia.

[27] Liu CC, Liu CC, Kanekiyo T, Xu H, Bu G (2013). Apolipoprotein E and Alzheimer disease: risk, mechanisms and therapy. Nat Rev Neurol.

[28] Hutzen B, Bid HK, Houghton PJ, Pierson CR, Powell K, Bratasz A (2014). Treatment of medulloblastoma with oncolytic measles viruses expressing the angiogenesis inhibitors endostatin and angiostatin. BMC Cancer.

[29] He J, Xiao H, Li B, Peng Y, Li X, Wang Y (2019). The programmed site-specific delivery of the angiostatin sunitinib and chemotherapeutic paclitaxel for highly efficient tumor treatment. J Mater Chem B.

[30] Sando SB, Melquist S, Cannon A, Hutton ML, Sletvold O, Saltvedt I (2008). APOE epsilon 4 lowers age at onset and is a high risk factor for Alzheimer’s disease; a case control study from central Norway. BMC Neurol.

[31] Li WW, Shen YY, Tian DY, Bu XL, Zeng F, Liu YH (2019). Brain Amyloid-beta deposition and blood biomarkers in patients with clinically diagnosed Alzheimer’s disease. J Alzheimer’s Dis: JAD.

[32] Carandini T, Arighi A, Sacchi L, Fumagalli GG, Pietroboni AM, Ghezzi L (2019). Testing the 2018 NIA-AA research framework in a retrospective large cohort of patients with cognitive impairment: from biological biomarkers to clinical syndromes. Alzheimer’s Res Ther.

