# Supplementary material for: Associations of plasma angiostatin and amyloid-β and tau levels in Alzheimer’s disease
Source: Transl Psychiatry. 2022 May 10;12:194. doi: 10.1038/s41398-022-01962-6 (PMC9091258; doi:10.1038/s41398-022-01962-6)
Supplement: Supplementary file 1 — Supplemental Information [file 41398_2022_1962_MOESM1_ESM.docx]

**Supplemental Information**

**Associations of plasma angiostatin and amyloid-β and tau levels in Alzheimer's disease**

Yuan Cheng^1,2,3^, Jun-Rong Ren^1,2,3^, Jie-Ming Jian^1,2,3^, Chen-Yang He^1,2,3^, Man-Yu Xu^1,2,3^, Gui-Hua Zeng^1,2,3^, Cheng-Rong Tan^1,2,3^, Ying-Ying Shen^1,2,3^, Wang-Sheng Jin^1,2,3^, Dong-Wan Chen^1,2,3^, Hui-Yun Li^1,2,3^, Xu Yi^1,2,3^, Yuan Zhang^1,2,3^, Xian-Le Bu^1,2,3,*^, Yan-Jiang Wang^1,2,3,4,*^

1. Department of Neurology and Centre for Clinical Neuroscience, Daping Hospital, Third Military Medical University, Chongqing, China.

2. Institute of Brain and Intelligence, Third Military Medical University, Chongqing, China.

3. Chongqing Key Laboratory of Ageing and Brain Diseases, Chongqing, China.

4. Center for Excellence in Brain Science and Intelligence Technology, Chinese Academy of Sciences, Shanghai, China.

* Correspondence to yanjiang_wang@tmmu.edu.cn (Y.J.W.), and buxianle@sina.cn (X.L.B.)

**Supplemental Table 1. Characteristics of the participants with CSF samples.**

| **Characteristics** |  | **CN (N=35)** | **AD dementia (N=25)** | ***p* values** |
| --- | --- | --- | --- | --- |
| Age, mean (SD), y |  | 67.46 (5.79) | 66.17 (10.75) | 0.5577 |
| Female, N (%) |  | 16 (45.71) | 12 (48.00) | 0.7889 |
| Education level, mean (SD), y |  | 9.31 (4.14) | 9.52 (4.37) | 0.8558 |
| MMSE score, mean (SD) |  | 26.88 (2.07) | 13.28 (6.91) | <0.0001 |
| APOE-ε4 carriers, N (%) |  | 8 (22.86) | 13 (52.00) | 0.0127 |
| Diabetes (%) |  | 4 (11.43) | 4 (16.00) | 0.7001 |
| Hypertension (%) |  | 6 (17.12) | 5 (20.00) | 0.7385 |
| Coronary artery disease (%) |  | 8 (22.86)) | 6 (24.00) | >0.9999 |
| Stroke history (%) |  | 3 (8.57) | 2 (8.00) | >0.9999 |

Results are shown as mean (SD) or number (%). Abbreviations: CN, Cognitively normal control; AD, Alzheimer’s disease; y, years; MMSE, Mini-Mental State Examination; APOE, apolipoprotein E; N, number; SD, standard deviations; CSF, cerebrospinal fluid.

**Supplemental figure 1.**


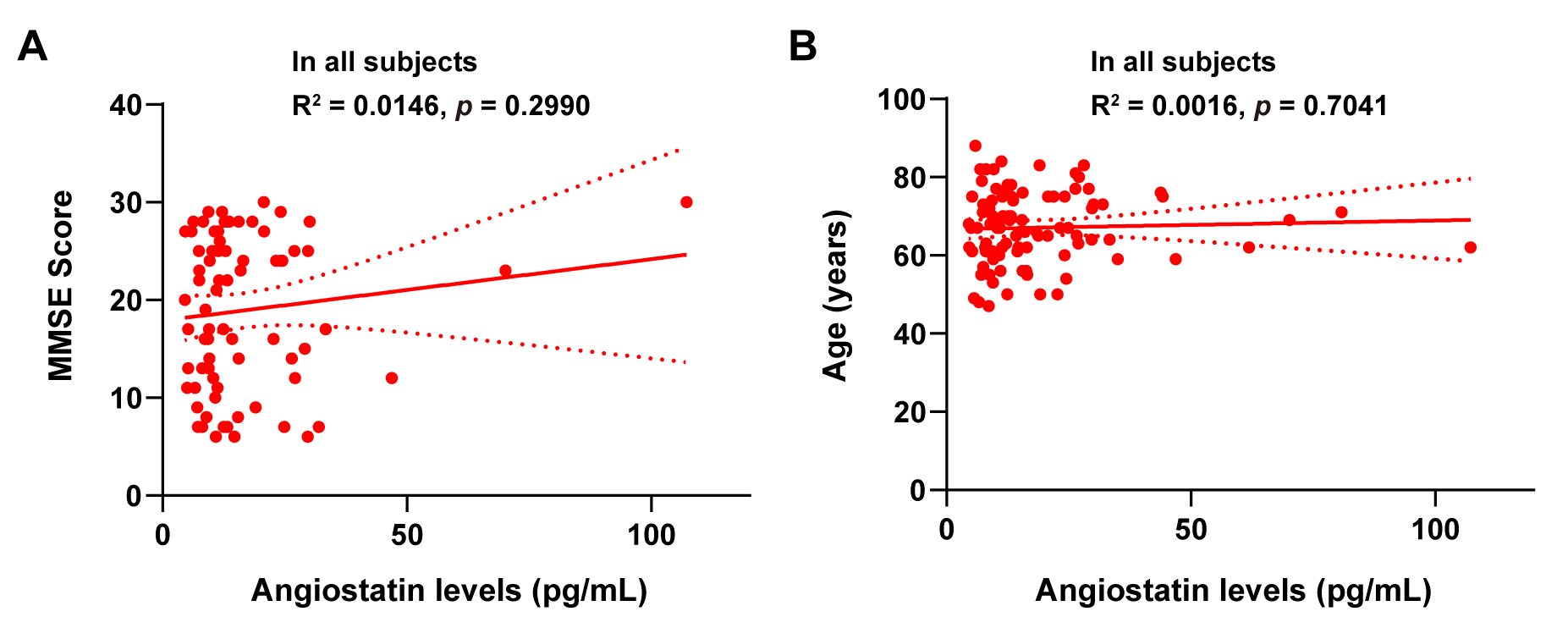


**Supplemental figure 1. The correlation analyses of plasma angiostatin with MMSE score and age in all subjects.** Correlations of angiostatin levels with MMSE score (A). Correlations of angiostatin levels with age (B).
